# An updated meta-analysis of amantadine for treating dyskinesia in Parkinson's disease

**DOI:** 10.18632/oncotarget.17622

**Published:** 2017-05-05

**Authors:** Min Kong, Maowen Ba, Chao Ren, Ling Yu, Shengjie Dong, Guoping Yu, Hui Liang

**Affiliations:** ^1^ Department of Neurology, Yantaishan Hospital, Yantai City, Shandong 264000, PR China; ^2^ Department of Neurology, The Affiliated Yantai Yuhuangding Hospital of Qingdao University, Shandong 264000, PR China

**Keywords:** dyskinesia, amantadine, meta-analysis

## Abstract

In recent years, a few of randomized controlled trials (RCTs) about amantadine for treating dyskinesia in Parkinson's disease (PD) were completed. Here, we conducted a systematic literature review about the clinical research to provide the updated evidence for treating dyskinesia. Electronic search of Medline, PubMed, Cochrane Library, and other databases up to May 2016 for relevant studies was performed. We selected the Unified Parkinson's Disease Rating Scale IV (UPDRS IV) and Dyskinesia Rating Scales (DRS) as efficacy outcomes of amantadine on dyskinesia. Pooled data from included studies was then used to carry out meta-analysis. A total of eleven eligible RCTs that involved 356 PD patients with existing dyskinesia were included in the present study. The results of meta-analysis showed that amantadine significantly improved UPDRS IV (*P* < 0.0001) and DRS (*P* < 0.00001). Meanwhile, there was a mild reduction in Unified Parkinson's Disease Rating Scale III after amantadine treatment in advanced PD patients with dyskinesia (*P* = 0.01) compared with placebo. High dosage of amantadine obviously improved existing dyskinesia in PD, yet at the expense of the increased adverse events. It was necessary to detect the optimal therapeutic efficacy to balance the incidence of adverse events when we used amantadine to treat existing dyskinesia in PD patients.

## INTRODUCTION

Parkinson's disease (PD) is one of age-related neurodegenerative diseases with bradykinesia, resting tremor, rigidity, posture and gait instability. As we all know, levodopa, the dopamine precursor, is the most effective drug for treating PD. Unfortunately, after five to ten years of levodopa replacement treatment, most of PD patients are troubled with disabling dyskinesia, which presents abnormal involuntary movements in trunk, head and extremities, and thus severely impacts daily life of PD patients [1, 2].

There were evidences for changes in glutamatergic markers in PD patients. Evidences also suggest that dyskinesia is at least partly associated with abnormal striatal glutamatergic overactivity due to pathological interaction between dopamine and glutamate inputs [3, 4]. On this point, overactivity of striatal glutamatergic *N*-methyl-*D*-aspartate receptor (NMDAR) has been implicated in the pathogenesis of PD and dyskinesia from current research including our research reports. Thus, these pathological molecular events can also become available targets for treating dyskinesia. Indeed, in preclinical animal research of dyskinesia, the antagonists of NMDAR have demonstrated good therapeutic effects [5–7].

In clinic, amantadine is one drug for treating PD in the early stage of disease. Based on above mentioned evidences, as one noncompetitive antagonist of NMDAR, amantadine can also benefit for treating dyskinesia [8]. Thus, greater concentration was involved in the amantadine for treating dyskinesia by amelioration of glutamatergic neurotransmission. The researchers also conducted a series of clinical trials on amantadine for treating dyskinesia

Until today, as far as we know, only two systematic reviews concerning amantadine have been done to investigate the efficacy in dyskinesia by Elahi and Crosby [9, 10], who included several clinical trials with a small study population. The evidences for anti-dyskinetic effects of amantadine might not be sufficient. Therefore, the findings should be repeated in a larger study population. Recently, four more trials on dyskinesia in PD were completed, and not included in the previous reviews. Our meta-analysis included the recent data to access effects of amantadine in dyskinesia, and aimed to demonstrate a concise, clinically relevant summary for amantadine treating dyskinesia in PD.

## RESULTS

### Literature selection and study characteristics

**Figure 1:**
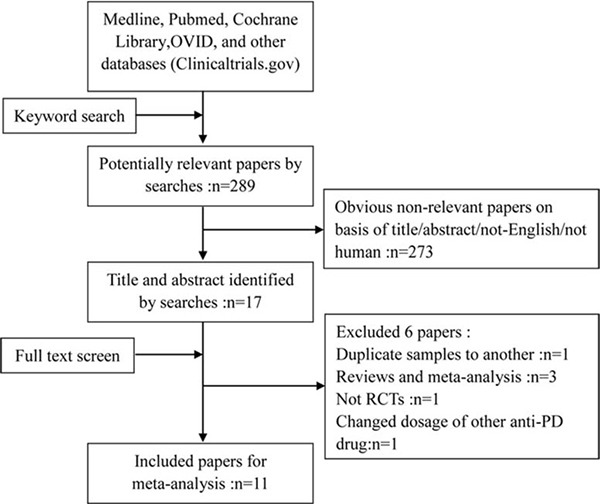
Flow chart of studies by screening, inclusion and exclusion

Finally, a total of eleven literatures fulfilled the inclusion criteria and were selected for meta-analysis [11–21]. The search strategy was demonstrated in Figure 1. The included literatures were published between 1998 and 2016. In addition, the included trials were all RCTs. Compared the final published data in 2004, one study was excluded due to the preliminary results [22]. One study was excluded because of not RCTs [23]. One study was excluded because of the changed dosage of other anti-PD drugs during the trials [24]. Three studies was excluded because of the reviews and meta-analysis type [9, 10, 25].

Among the included studies, there were seven randomized, parallel groups design and four randomized, cross-over design. The participants were diagnosed with PD for 7.9–16.8 years. The age of the participants in the trials was 59.7–67 years. All PD participants developed dyskinesia. The Unified Parkinson's Disease Rating Scale (UPDRS) Part IV or the Movement Disorder Society Unified Parkinson's Disease Rating Scale (MDS-UPDRS) Part IV as the outcome measure of dyskinesia was observed in nine studies. Various dyskinesia rating scale (DRS) as the outcome measure of dyskinesia was observed in ten studies. These DRS included abnormal involuntary movements scale (AIMs), clinical dyskinesia rating scale (CDRS), Marconi dyskinesia rating Scale (Marconi DRS), Goetz dyskinesia rating Scale (Goetz DRS), unified dyskinesia rating scale (UDysRS), and rush dyskinesia rating scale (RDRS). We only measured the immediate outcome of dyskinesia after the last dose of medication used in each study due to obvious different follow-up period (range 0–12 months). The dosage and treatment duration of amantadine varied in different trials. The duration of amantadine administration varied from three hours to three month. In a four-arm EASED study by Pahwa [21], the primary efficacy analysis compared the 340-mg dose level of amantadine with placebo. Thus, we selected two-arm 340-mg dose level and placebo into the meta-analysis. A total of 356 PD patients with existing dyskinesia were included in the present study. The total number of dropout patients was 32. Data details of the included trials were demonstrated in Tables 1 and 2.

**Table 1:** Characteristics of the studies included in the meta-analysis

Trial	Design	Dosage	Follow up	Enrolment	Participants	Outcomes	Safety
Verhagen Metman 1998	CO	350 ± 15 mg/day	6 weeks	18	PD patients with peak-dose dyskinesias, H&Y stages 3.5 ± 0.2	UPDRS IV AIMs	AEs
Luginger 2000	CO	300 mg/day	2 weeks	11	PD patients with peak-dose dyskinesias, H&Y stages 2.8 ±1.2	UPDRS III, IV Marconi DRS	AEs
Snow 2000	CO	100–200 mg/day	3 weeks	24	PD patients with dyskinesias, H&Y stages (-)	UPDRS III, IV Goetz DRS	AEs
Del Dotto 2001	P	200 mg IV	3 hours	9	PD patients with peak-dose dyskinesias, H&Y stages 3.0 ± 0.5	UPDRS III AIMs	AEs
Thomas 2004	P	300 mg/day	15 days	40	PD patients with peak-dose or biphasic dyskinesias, H&Y stages 2.6 ± 0.2	UPDRS III, IV Goetz DRS	AEs
Silva-Junior 2005	P	100–200 mg/day	3 weeks	18	PD patients with peak-dose dyskinesias, H&Y stages 2.5 ± 0.5	UPDRS III, IV CDRS	AEs
Wolf 2010	P	100 mg/day	3 weeks	32	PD patients with peak-dose dyskinesias, H&Y stages (−)	UPDRS III, IV	AEs
Sawada 2010	CO	300 mg/day	27 days	35	PD patients with peak-dose dyskinesias, H&Y stages (−)	UPDRS III, IV RDRS	AEs
Goetz 2013	P	300 mg/day	8 weeks	68	PD patients with peak-dose dyskinesias, H&Y stages 2	UDysRS	AEs
Ory-Magne 2014	P	≥ 200 mg/day	3 month	56	PD patients with peak-dose dyskinesias, H&Y stages (−)	UPDRS III, IV AIMs	AEs
Pahwa 2016	P	340 mg/day	8 weeks	43	PD patients with peak-dose dyskinesias, H&Y stages 2.5 ± 0.7	MDS-UPDRS IV UDysRS	AEs

All trials included were randomised, double-blind, placebo-controlled trials.

CO, Cross over; P, Parallel design; PD, Parkinson's disease; UPDRS, Unified Parkinson's Disease Rating Scales; AIMs: Abnormal Involuntary Movements Scale; CDRS: Clinical Dyskinesia Rating Scale; DRS: Dyskinesia Rating Scale; UDysRS, Unified Dyskinesia Rating Scale; RDRS, Rush Dyskinesia Rating Scale; MDS-UPDRS, the Movement Disorder Society Unified Parkinson's Disease Rating Scale; H&Y: Hoehn and Yahr Parkinson's disease staging scale; AEs, Adverse Events; H&Y: Hoehn and Yahr Parkinson's disease staging scale; Follow-up indicates the most immediate evaluation time point after the end of treatment for each study. This is different from the maximum follow-up time for each study.

**Table 2:** Characteristics of patients in dyskinesia trials

Trial	Patients (Drug/Placebo)	Age years	Duration of PD	H&Y	UPDRS IV		Dyskinesia		UPDRS III	
					Drug	Placebo	Drug	Placebo	Drug	Placebo
Verhagen Metman 1998	18 (14/14) (4 dropout)	60 ± 2	13 ± 1.3	3.5 ± 0.2	1(Items32, 34, 39)	4(Items32, 34, 39)	3.6 ± 2.25 (AIMs)	7.0 ± 3.38 (AIMs)	NA	NA
Luginger 2000	11 (10/10) (1 dropout)	63.5 ± 8.2	10.1 ± 5.1	2.8 ±1.2	7.0 ± 8.2 (IVa)	14.5 ± 9.4 (IVa)	9.1 ± 9.1 (DRS)	19.3 ± 13.7 (DRS)	50 ± 20	68 ± 20
Snow 2000	24 (22/22) (2 dropout)	64.2 ± 8.9	10.6 ± 3.6	NA	3.2 ± 1.6 (IVa)	4.3 ± 1.5 (IVa)	22.0 ± 13.2 (DRS)	29.0 ± 12.6 (DRS)	22.3 ±12.1	23.4 ±9.0
Del Dotto 2001	9 (5/4) (0 dropout)	59.7 ± 8	8.4 ± 3.0	3.0 ±0.5	NA	NA	4.1 ± 1.7 (AIMs)	8.3 ± 1.8 (AIMs)	21.6 ± 9.5	23.5 ± 9.7
Thomas 2004	40 (17/18) (5 dropout)	62.7 ± 5.2	7.9 ±2.2	2.6 ±0.2	2.0 ± 1.1 (IVa)	6.1 ± 2.6 (IVa)	10.5 ± 1.3 (DRS)	20.2 ±1.6 (DRS)	48.1 ± 7.8	52.5 ± 8.3
Silva-Junior 2005	20 (9/9) (2 dropout)	60.6 ± 9.8	8.9±3.8	2.5±0.5	2.8±2.1 (IVa)	3.7±1.8 (IVa)	6.8±4.9 (CDRS)	13.0±11.5 (DRS)	16.3±9.3	18.7±5.3
Wolf 2010	32 (14/17) (1 dropout)	67±7.7	16.8±5.9	NA	3.6±0.4 (Items32, 33)	4.4±0.4 (Items32, 33)	NA	NA	25.8±3.4	27.7±3.7
Sawada 2010	35 (30/32) (5 dropout)	63.9±7.6	13.5±4.5	NA	5.87±3.6 (IVa)	7.73±3.1 (IVa)	1.1±0.7 (RDRS)	2.1±0.8 (RDRS)	18.32±14.0	18.12±8.6
Goetz 2013	68 (36/32) (7 dropout)	65.4±8.2	9.0±3.5	Median 2 (1-4)	NA	NA	20.71 ± 8.89 (UDysRS)	34.07 ± 12.51 (UDysRS)	NA	NA
Ory-Magne 2014	56 (27/29) (0 dropout)	64.0±7.7	13.6±6.7	NA	3.3±1.7 (Items32, 33)	4.9±1.5 (Items32, 33)	2.4 ± 2.8 (AIMs)	5.7 ± 2.5 (AIMs)	16.0±8.1	17.0±8.2
Pahwa 2016	43 (21/22 ) (5 dropout)	66.0±9.5	9.5±5.0	2.5±0.7	9.3±2.8 ( IV)	11.7±3.1 (IV)	25.9 ± 12.1 (UDysRS)	32.5 ± 17.8 (UDysRS)	NA	NA

AIMS: Abnormal Involuntary Movements Scale; CDRS: Clinical Dyskinesia Rating Scale; DRS: Dyskinesia Rating Scale; UPDRS, Unified Parkinson's Disease Rating Scales; H&Y: Hoehn and Yahr Parkinson's disease staging scale; UDysRS, Unified Dyskinesia Rating Scale; RDRS, Rush Dyskinesia Rating Scale.

**Figure 2:**
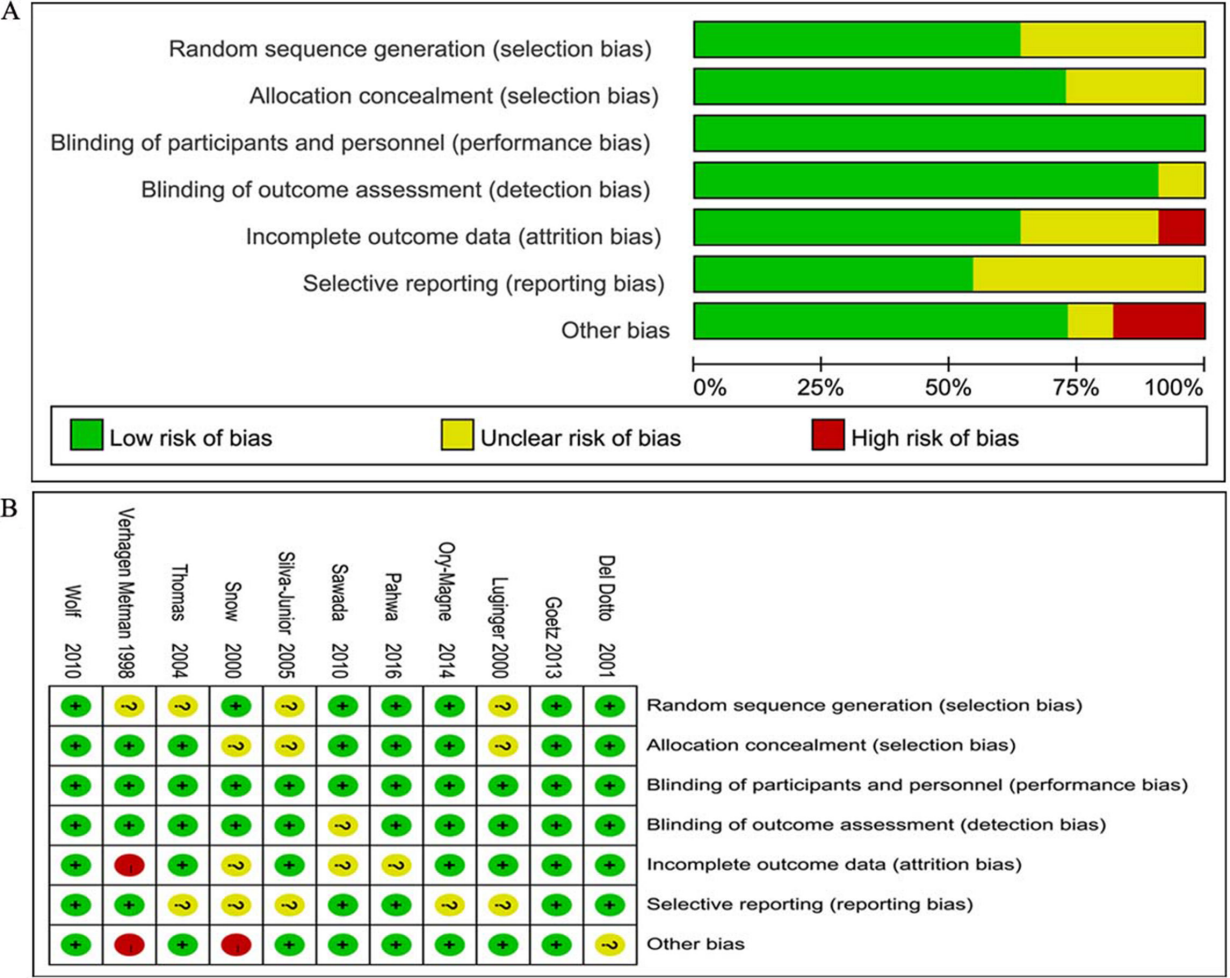
Bias risk assessment (**A**) Risk of bias graph. (**B**) Risk of bias summary.

**Figure 3:**
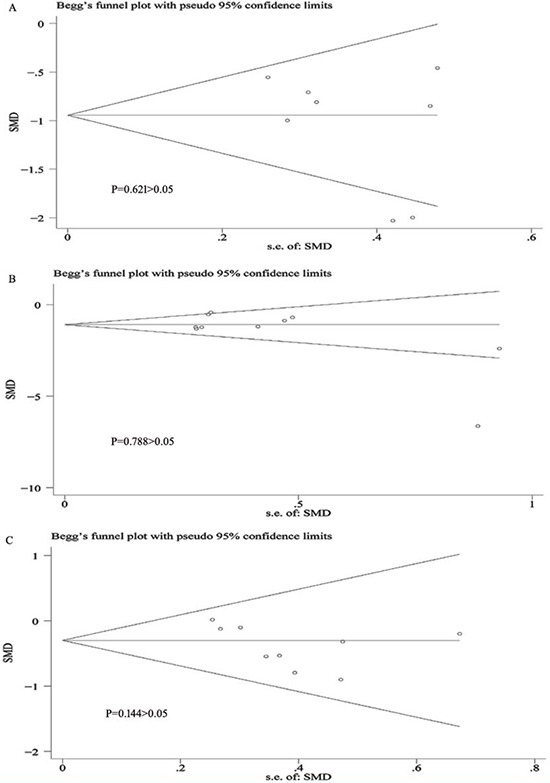
Bias assessment plot for the effect of amantadine on UPDRS IV (**A**), DRS (**B**) and UPDRS III (**C**) score by funnel blot and Begg's test.

Cochrane Handbook for Systematic Reviews was used to assess the risk of bias in the eleven included literatures. Though all included trials stated randomization, 7 trials showed the means of random sequences generation (for example, computer generated, random number generator). 8 trials presented the message about an appropriate concealment allocation. All the trials showed the blinding of participants. Incomplete outcome data was only found in one trial. Six trials were non-selective reporting, and the other five trials were uncertain. Two trials existed certain degree other potential threats to validity. Thus, all the included trials were believed to have a low bias risk (Figure 2). The funnel plots for the study of amantadine showed low likelihood of publication bias by Begg's test for on UPDRS IV(*P* = 0.621), DRS (*P* = 0.788) and UPDRS III (*P* = 0.144) (Figure 3).

### Efficacy outcomes

**Figure 4:**
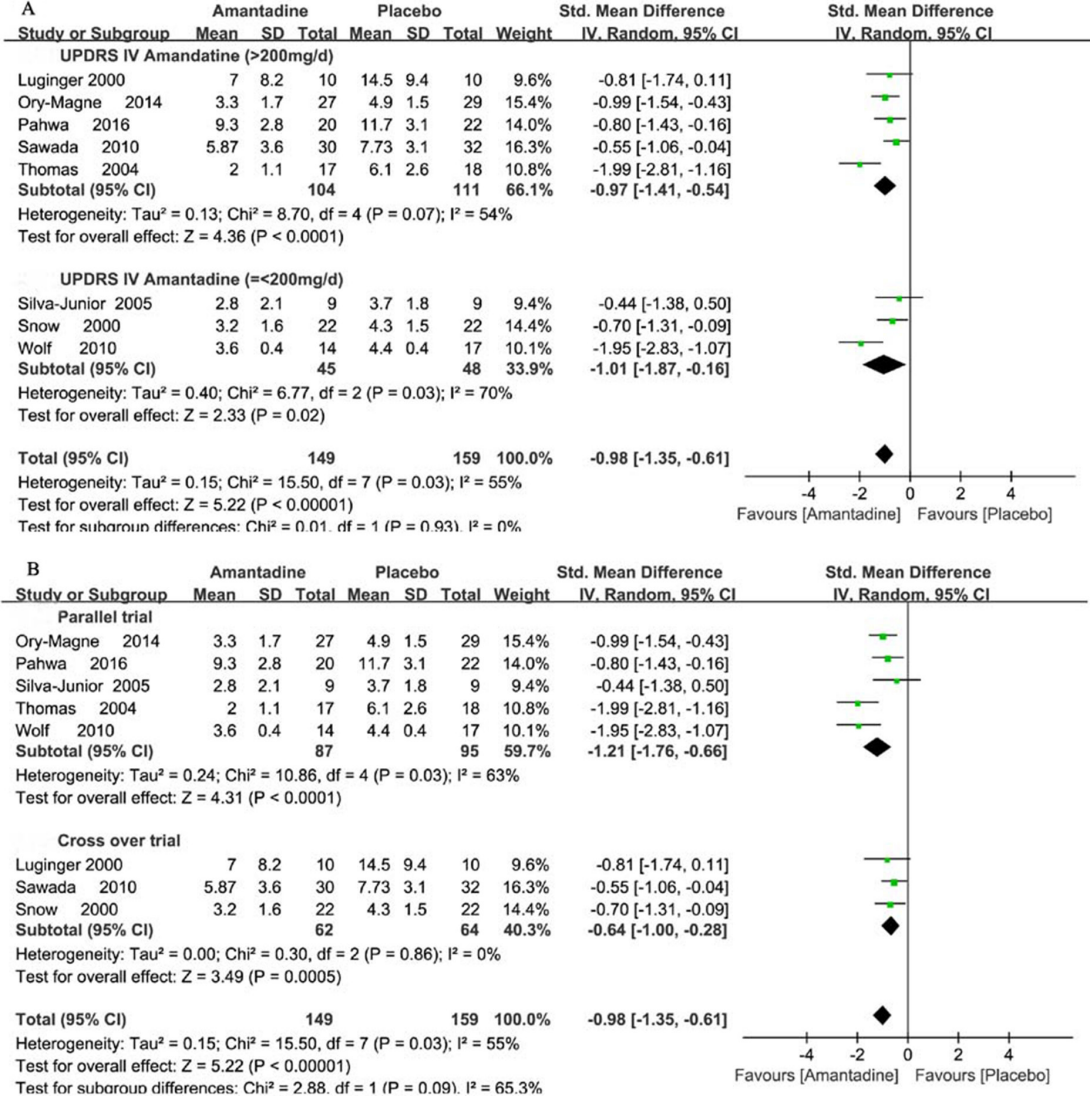
Forest plot of dyskinesia assessment comparison on UPDRS IV in amantadine and placebo by drug dosage and trial design

UPDRS IV as the outcome measure of dyskinesia was observed in nine studies. One trial reported the outcome of UPDRS IV as the median form. So, only eight trials reported the detailed outcome of UPDRS IV. In meta-analysis, amantadine produced significant effects on UPDRS IV scores, and SMDs were −0.98 points (95% CI −1.35 to −0.61, *P* < 0.00001) compared with placebo. In general, the meta-analysis for amantadine demonstrated mild heterogeneity with I^2^ = 55% (*P* = 0.03). The one study by Verhagen Metman failed to pool analysis due to the original data demonstrated in the form of median improvement [11], but it reported the significant effects of amantadine for improving the UPDRS IV compared with the placebo group (*P* < 0.01). In addition, in view of the difference of amantadine dosage and trial design in each trial, subgroup analysis of UPDRS IV for different dosage of amantadine showed that compared with placebo, in high dosage of amantadine, SMDs were −0.97 points (95% CI −1.41 to −0.54, *P* < 0.00001) with heterogeneity of I^2^ = 54% (*P* = 0.07) and in low dosage of amantadine, SMDs were −1.01 points (95% CI −1.87 to −0.16, *P* = 0.02) with heterogeneity of I^2^ = 70% (*P* = 0.03). Subgroup analysis of heterogeneity for different trial design showed that in parallel trials, heterogeneity showed I^2^ = 63% (*P* = 0.03) and in cross over trial, heterogeneity showed I^2^ = 0% (*P* = 0.86) (Figure 4).

**Figure 5:**
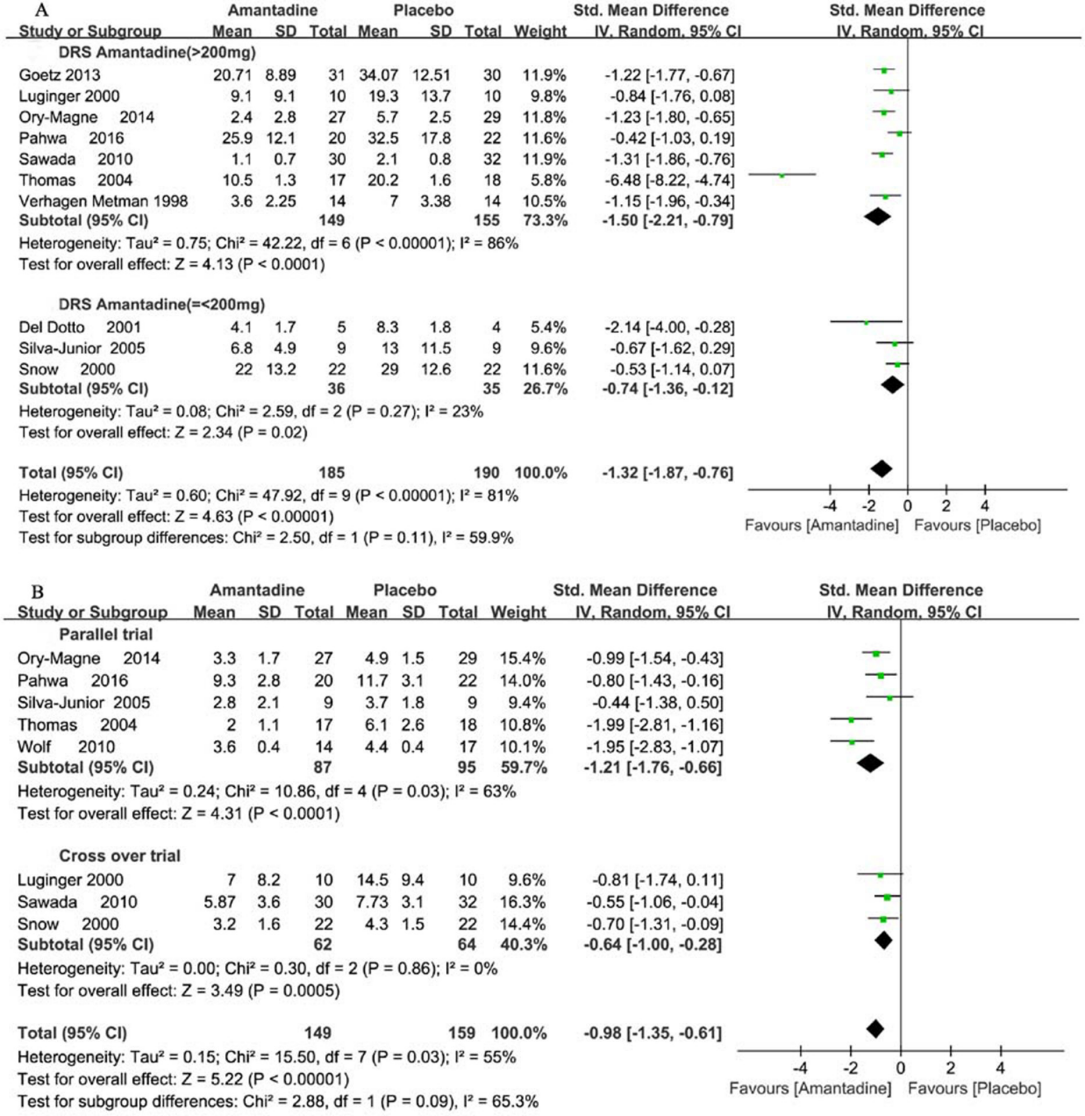
Forest plot of dyskinesia assessment comparison on DRS in amantadine and placebo by drug dosage and trial design

Ten trials reported the detailed outcome of DRS. In meta-analysis, amantadine produced significant effects on DRS scores, and SMDs were −1.32 points (95% CI −1.87 to −0.76, *P* < 0.00001) compared with placebo. In general, the meta-analysis for amantadine demonstrated significant heterogeneity with I^2^ = 81%. Subgroup analysis for different dosage of amantadine showed that in high dosage of amantadine, SMDs were −1.5 points (95% CI −2.21 to −0.79, *P* < 0.0001) with heterogeneity of I^2^ = 86% (*P* = 0.00001), and in low dosage of amantadine, SMDs were -0.74 points (95% CI −1.36 to −0.12, *P* = 0.02) with heterogeneity of I^2^ = 23% (*P* = 0.27) compared with placebo. Subgroup analysis of heterogeneity in parallel and cross over trials showed heterogeneity with I^2^ = 89% (*P* < 0.00001) and 20% (*P* = 0.29), respectively (Figure 5).

**Figure 6:**
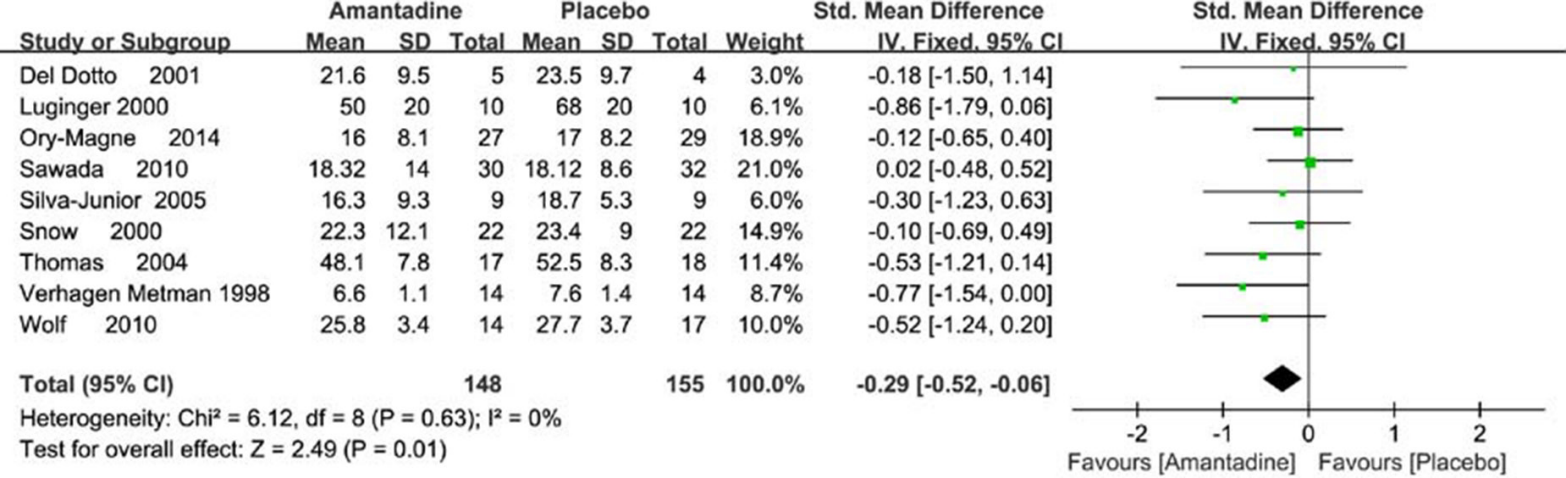
Forest plot of PD motor symptoms assessment comparison on UPDRS III in amantadine and placebo

Nine trials reported the detailed outcome of UPDRS III. In meta-analysis, amantadine produced significant effects on UPDRS III scores, and SMDs were −0.29 points (95% CI −0.52 to −0.06, *P* = 0.01) compared with placebo. In general, the meta-analysis for amantadine demonstrated no significant heterogeneity with I^2^ = 0% (Figure 6).

### Adverse effects

**Figure 7:**
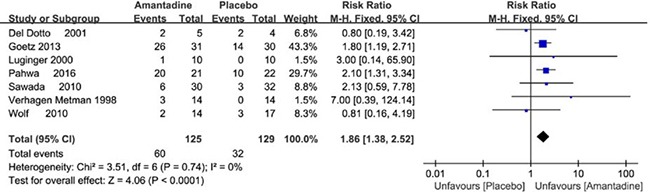
Forest plot of safety outcomes comparison on adverse events (AEs) in amantadine and placebo

Available data on adverse effects (AEs) were mentioned in seven trials. Amantadine in general demonstrated statistically obvious higher rates of AEs than placebo (RR 1.85 95% CI 1.39 to 2.46, *P* < 0.0001). The common AEs included visual hallucinations, confusion, blurred vision, feet edema, constipation and so on. High dosage of amantadine demonstrated more obviously higher rates of AEs than placebo (RR 1.97 95% CI 1.46 to 2.65, *P* < 0.0001). However, there was no obvious discrepancy of AEs between the low dosage of amantadine and placebo. (RR 0.8 95% CI 0.27 to 2.39, *P* = 0.69) (Figure 7). The AEs in the AMANDYSK trial by Ory-Magne et al [20] were not included in the meta-analysis for we couldn't distinguish whether the AEs were caused by placebo or the discontinued amantadine.

## DISCUSSION

Based on the current meta-analysis, amantadine could evidently improve the UPDRS IV and DRS score compared with placebo in PD patients with dyskinesia. Meanwhile, amantadine can also mildly improve UPDRS III, one marker of motor symptoms of PD. These results displayed that amantadine can not only reduce dyskinesia in PD patients, but also benefit parkinsonian symptoms even in advanced stage of PD. However, the results of the study demonstrated obvious adverse effects in amantadine treatment compared with the placebo, especially at high dosage of amantadine, such as visual hallucinations, confusion, blurred vision, feet edema, constipation and so on. Hence, it was necessary to detect the optimal therapeutic efficacy to balance the incidence of adverse events when we used amantadine for treating dyskinesia.

Dyskinesia is abnormal involuntary movements mainly involving the extremities, trunk, or jaw. In recent years, evidence suggests that the underlying mechanism of dyskinesia is closely associated with the changes in dopamine receptors and in the subunit phosphorylation pattern of co-existed ionotropic NMDA glutamatergic receptors. NMDAR sensitization can enhance cortical excitatory input to the striatal spiny efferent neurons, thus change striatal output and compromise motor functions [3–6]. As we all know, amantadine is used as an anti-PD drugs especially in the early stage of PD due to promoting dopamine release. Amantadine is also found to be noncompetitive antagonist of NMDAR which played a pivotal role in the pathogenesis of dyskinesia [9]. Blockade of NMDAR by NMDAR antagonists, amantadine, can block glutamatergic transmission and regulate corticostriatal synaptic efficacy [25, 26]. In addition, amantadine can increase striatal neo-synthesis D2 receptors in rats which represent one reinforcing mechanism of drug efficacy [27]. Based on such finding, amantadine has been shown to reduce the severity of existing dyskinesia in advanced PD patients. Our meta-analysis further confirmed this point. Yet, we can't draw conclusions whether amantadine can reduce the development of dyskinesia in PD patients without motor complications. Therefore, we should interpret the result prudently.

In addition, we carried out quality assessment according to the Cochrane Handbook for Systematic Reviews of Interventions, and the qualities of the evidence reached high levels. The strength of this meta-analysis included the recently published four RCTs [18–21], which were not included in previous review. Yet, we found significant heterogeneity of these included RCTs. So, several limitations of the study could still exist. First, some items tested in the trials were not available in the results, and despite numerous attempts to contact the authors, further details were still absent. Second, the different dosage of amantadine administration and trial design may also partly reduce the precision of our findings as reflected in subgroup analysis in the present study. The dosage of amantadine varied greatly. Two cross-over trials had no wash-out interval between the treatment periods [11, 13]. There could be the risk of a carry-over effect into the second arm. Third, trials with different treatment duration were allowed in this study, which could affect the efficacy and safety assessment. Moreover, a large proportion of the studies included in this review are less than three months in duration. There are insufficient data on the comparative efficacy and tolerability of amantadine beyond three months. Only in one study patients on stable doses of amantadine for at least one year were randomized to receive placebo or continue taking amantadine. This study reported worsening of symptoms after amantadine cessation and demonstrated longer term effects of amantadine therapy [17]. Dyskinesia in PD patients can persist a relative long term. It was very important to know if antidyskinetic actions of amantadine persist for a longer period. Fourth, the sample size was small in several trials. Various DRS scale, baseline condition of PD patients in DRS score, variability in the PD participants and other combination anti-PD drug therapy could potentially affect this meta-analysis. Therefore, it is necessary to carry out more strict RCTs with larger sample and long duration to assess the efficacy of amantadine in PD patients with dyskinesia. Finally, we only analyzed published study in English which could lead to a publication bias for favorable results.

So far, only two systematic reviews have been published on this topic by Elahi and Crosby [9, 10]. After including four recent studies, the present meta-analysis of RCTs mainly focused on updating the efficacy of amantadine for treating dyskinesia in PD patients, and added to subgroup analysis and AEs assessment. In spite of understanding the limitations of the meta-analysis, our findings still demonstrate many high-quality RCTs and provide effective evidences that amantadine can benefit for dyskinesia at least in a relative short term in PD patients with dyskinesia. Further RCTs on a larger scale are still needed to better evaluate long-term efficacy and safety of amantadine on dyskinesia.

## MATERIALS AND METHODS

### Search strategy

We searched Medline, Pubmed, Cochrane Library, and other databases (Clinicaltrials.gov) up to May 2016 for all English language publications. Reference lists from the resulting reviews and publications were used to identify further relevant publications. The following search terms used were: amantadine, Parkinson Disease, Parkinson's Disease, Parkinsonism, PD, Paralysis Agitans, motor complications, and dyskinesia. The following was Pubmed (Medline) search strategy, which was modified to suit Cochrane Library database.

1. Dyskinesia

2. Motor complications

3. OR/1–2

4. Parkinson's disease

5. Parkinson disease

6. Parkinsonism

7. PD

8. Paralysis Agitans

9. OR/7–8

10. Amantadine

11. 3 AND 9 AND 10

### Selection criteria

The prospective randomized controlled trials (RCTs) assessing amantadine with placebo for treating dyskinesia in PD patients were included in our meta-analysis. The included patients must fulfill standard diagnostic criteria for PD according to the UK Parkinson's Disease Society Brain Bank (UKBB) criteria or clinically probable and definite PD diagnosis [28], and had developed levodopa-induced dyskinesia. There was a stable drug medication for one month before the trial and throughout the study. The eligible studies could provided the detailed data, such as randomized patients number, main outcome measures, amantadine medication formulations and doses, trial duration, double-blinding and randomization.

### Data extration

Two authors extracted data from each study independently, including trial design, first author, year of publication, numbers randomized (amantadine and placebo), mean age, PD duration, Hoehn and Yahr (H&Y) stages, amantadine medication formulations and doses, trial durations, blinding, main outcome measures, adverse events. If the trial was comparing different dosages of amantadine versus control, then the arm using generally recommended dosage was chosen for inclusion in the analysis. We resolved the disagreements by discussion with the third author. We would try to contact the author to get more information or calculated by ourselves based on the Cochrane Handbook if the data for meta-analysis were missing or only expressed graphically. If need, we would try to contact pharmaceutical companies to get necessary data. We evaluated the the risk bias of RCTs in line with the Cochrane Handbook for Systematic Reviews of Interventions [29].

### Data analysis

The standardized mean differences (SMD) in continuous outcomes and risk ratios (RR) in dichotomous variables with 95% CI and *P* values were calculated to assess effects of study drugs. In meta-analysis, SMD is applied as an aggregated statistics when all trials evaluated the same outcome, but assessed it with many kind of methods (such as different rating scales). In this circumstance, it is necessary to standardize the result for different kind of dyskinesia rating scale in the included literature. We used the inverse variance method in continuous variables with random effects model and/or fixed effects model to combine data and generate the overall effect estimate according the degree of heterogeneity. The degree of heterogeneity was assessed by a χ^2^ test combined with the I^2^ method (I^2^ < 25% representing low heterogeneity, and I^2^ > 75% representing high heterogeneity). High heterogeneity is modeled with random effects, and vice versa with fixed effect models. Subgroup analysis for the different trial design, different dosage of amantadine and different assessment methods were performed to examine methodological variations among studies and exclude the study that may bias the combined results with the rest studies being recalculated. The analysis was performed with Revman version 5.1. *P* < 0.05 represents statistically significant. Funnel plotting and Begg's test were used to assess publication bias with Revman version 5.1 and Stata version 12.0.
